# Antipyrin in Sunstroke

**Published:** 1885-09

**Authors:** 


### Antipyrin in Sunstroke.

Dr. Benj. F. Westbrook, of Brooklyn, in the *New York Medical Journal*, reports the treatment of two cases of sunstroke with high temperature by antipyrin hypodermically. Both cases recovered, but were of the class that usually die, the temperature in rectum being respectively 109° and 110° when first seen.

In the first case, one drachm of antipyrin, given hypodermically in two doses, reduced the temperature in one hour and a quarter to 99° in the rectum. Thirty grains hypodermically, in the second case, reduced the temperature to 101° in thirty-five minutes.

Chloral, bromides, quinine and stimulants were given in both cases as indicated. Dr. Westbrook says their prompt recovery was miraculous almost, and he believes the antipyrin the drug that saved their lives.

